# Heat Adaptation Induced Cross Protection Against Ethanol Stress in *Tetragenococcus halophilu*s: Physiological Characteristics and Proteomic Analysis

**DOI:** 10.3389/fmicb.2021.686672

**Published:** 2021-06-18

**Authors:** Huan Yang, Shangjie Yao, Min Zhang, Chongde Wu

**Affiliations:** ^1^College of Biomass Science and Engineering, Sichuan University, Chengdu, China; ^2^Key Laboratory of Leather Chemistry and Engineering, Ministry of Education, Sichuan University, Chengdu, China

**Keywords:** *Tetragenococcus halophilus*, cross protection, ethanol stress, heat preadaptation, membrane properties, proteomic analysis

## Abstract

Ethanol is a toxic factor that damages membranes, disturbs metabolism, and may kill the cell. *Tetragenococcus halophilus*, considered as the cell factory during the manufacture of traditional fermented foods, encounters ethanol stress, which may affect the viability and fermentative performance of cells. In order to improve the ethanol tolerance of *T. halophilus*, a strategy based on cross protection was proposed in the current study. The results indicated that cross protection induced by heat preadaptation (45°C for 1.5 h) could significantly improve the stress tolerance (7.24-fold increase in survival) of *T. halophilus* upon exposure to ethanol (10% for 2.5 h). Based on this result, a combined analysis of physiological approaches and TMT-labeled proteomic technology was employed to investigate the protective mechanism of cross protection in *T. halophilus*. Physiological analysis showed that the heat preadapted cells exhibited a better surface phenotype, higher membrane integrity, and higher amounts of unsaturated fatty acids compared to unadapted cells. Proteomic analysis showed that a total of 163 proteins were differentially expressed in response to heat preadaptation. KEGG enrichment analysis showed that energy metabolism, membrane transport, peptidoglycan biosynthesis, and genetic information processing were the most abundant metabolic pathways after heat preadaptation. Three proteins (GpmA, AtpB, and TpiA) involved in energy metabolism and four proteins (ManM, OpuC, YidC, and HPr) related to membrane transport were up-regulated after heat preadaptation. In all, the results of this study may help understand the protective mechanisms of preadaptation and contribute to the improvement of the stress resistance of *T. halophilus* during industrial processes.

## Introduction

*Tetragenococcus halophilus*, a moderate halophilic Gram-positive lactic acid bacterium, has been widely applied to produce Chinese horsebean-chili-paste, soy sauce, and mucor-type Douchi (He et al., 2016a; Jeong et al., 2017). This halophilic lactic acid bacterium was reported to be one of the dominant microorganisms, which played a significant role in the production of pickled fermented products due to its high ability to promote the generation of volatile compounds and improve the flavor characteristics. For example, it had been demonstrated that the addition of *T. halophilus* in fish sauce fermentation improved amino acid profiles and volatile compounds as well as reduced biogenic amine content (Udomsil et al., 2011). In addition, it was also reported that a co-culture of *T. halophilus* and *Zygosaccharomyces rouxii* exhibited mutually beneficial interactions and contributed to improving the quality of soy sauce through the increase of volatile compounds and flavor characteristics (Devanthi et al., 2018; Yao et al., 2019). These studies indicated the potential of this species to be applied as a starter culture to improve the quality of fermented foods.

However, *T. halophilus* inevitably encountered multiple environmental stresses during the manufacture of these fermented foods, such as acid stress, thermal stress, osmotic stress, and ethanol stress (He et al., 2016a, 2017b; Yao et al., 2019), which would result in the decrease in enzyme activities as well as imbalance of cellular metabolisms. Therefore, investigation of the tolerance mechanisms and development of adaptive strategies to cope with various stresses were essential for cells during food fermentation (He et al., 2017a; Zhao et al., 2021). Previous research had reported that bacterial cells employed diverse approaches to adapt to harsh conditions, such as regulation of the membrane components and functions, up-regulation of the expression of stress response genes or proteins (He et al., 2017b; Guo et al., 2020). For instance, He et al. (2016a) suggested that *T. halophilus* responds to acid stress by adjusting intracellular pH and the NH_4_^+^ pool, the amount of amino acid (glutamate, aspartate, isoleucine, leucine, citrulline, ornithine), and regulating the expressions of classic stress response proteins (SSB, UspA, and GroL) and some proteins related to carbohydrate metabolism.

*Tetragenococcus halophilus* CGMCC 3792, originally isolated from soy sauce moromi in China, exhibits prominent tolerance to environmental stress conditions including salt, pH, heat and ethanol (Wu et al., 2013). However, high ethanol concentration would result in cell membrane injury, especially the plasma membrane, which directly leads to a decrease of membrane integrity and leakage of metabolites (Udom et al., 2019). In the past years, researchers successfully employed cross protection to improve the resistance to various environmental stresses (Olguin et al., 2015; He et al., 2016b; Huang et al., 2016; Zhao et al., 2021). For example, Huang et al. (2016) found that *Lactobacillus plantarum* ZDY2013 could improve the stress tolerance to 40 mM hydrogen peroxide and pH 3.0 by preadaptation at pH 4.5 for 2 h. A similar result reported that preadaptation at pH 3.5 for 1 h contributed to enhancing the resistance of *Alicyclobacillus acidoterrestris* to heat stress (65°C) (Zhao et al., 2021). Unfortunately, the protective mechanisms of cross protection in lactic acid bacteria had not been well understood. The current study aims to investigate the effect of cross protection induced by heat preadaptation on the tolerance of ethanol stress and the potential protective mechanisms in *T. halophilus* by physiological and proteomic analyses. Results presented in this study may contribute to systematically understanding the underlying mechanisms of cross protection and ethanol stress response of *T. halophilus*.

## Materials and Methods

### Strains and Growth Conditions

*Tetragenococcus halophilus* CGMCC 3792, originally isolated from soy sauce moromi, was used in this study (Wu et al., 2013). The bacterial strain was transferred from −80°C and incubated statically at 30°C in de Man, Rogosa, and Sharpe (MRS) broth (OXOID, United Kingdom).

### Cross Protection Experiments

Cells were harvested at the mid-exponential growth phase by centrifugation at 8000 × *g* for 5 min, washed with sterile water, and then resuspended in MRS. The resuspended cells were cultivated at 45°C for 1.5 h in 3 mL MRS broth with a concentration of 3 × 10^8^ CFU/mL for heat preadaptation (HP), and the cells without heat pretreatment were treated as control (BP). Cross protection experiments were performed by using cells treated with or without heat preadaptation (HP or BP). The HP and BP cells were subjected to heat, oxygen, salt, and ethanol stress, respectively. For heat stress, the HP and BP cells were exposed to 60°C in normal MRS for 2.5 h; For oxygen stress, the HP and BP cells were challenged at 0.03% H_2_O_2_ for 2.5 h; for salt stress, the HP and BP cells were subjected to 35% NaCl for 2.5 h; and for ethanol stress, the HP and BP cells were shocked by 10% ethanol for 2.5 h.

To investigate the exposure time of ethanol stress on the survival of *T. halophilus*, the HP and BP cells were then resuspended in MRS containing 10% ethanol at 30°C for 0, 1, 2.5, 4.5, and 6 h. Then, cells treated with or without heat preadaptation were challenged by 10% ethanol stress for 2.5 h, which were named HP-ES and ES, respectively. After ethanol stress, cell suspensions were centrifuged and washed with sterile PBS buffer (0.01 M, pH 7.0). The treated cells were plated onto MRS agar plates in triplicate and incubated at 30°C for 72 h to determine the survival ratio.

### Transmission Electron Microscopy Analysis

Cell membrane integrity was evaluated by transmission electron microscopy (TEM) analysis. Briefly, cells subjected to different treatments (BP, HP, HP-ES, and ES) were centrifuged and collected after washing twice with PBS buffer (0.01 M, pH 7.0). In total, 1 mL 2.5% glutaraldehyde was added to fix cells for 3 h at room temperature before being washed twice with PBS buffer (0.01 M, pH 7.0). Subsequently, the cells were washed, dehydrated, and embedded. Finally, ultrathin sections (70–80 nm) obtained by a Leica Ultracut UCT ultramicrotome (Leica) were captured using an electron microscope (JEM-1400, Japan) at room temperature.

### Scanning Electron Microscopy Analysis

The treated cells (BP, HP, HP-ES, and ES) were fixed with 2.5% glutaraldehyde for 3 h at room temperature, washed twice with PBS buffer (0.01 M, pH 7.0), and then dehydrated with different concentration gradients of ethanol for 3 min, respectively. The dehydrated cells were followed by specimen critical point drying to achieve absolute dry samples. After coating with gold-palladium for 2 min, cells were observed by a scanning electron microscope (JSM-7500F, JEOL, Tokyo, Japan).

### Atomic Force Microscopy Analysis

The treated cells (BP, HP, HP-ES, and ES) were collected and resuspended in 0.5 mL sterile water. In total, 10 μL suspension was transferred to a microstructure and dried at room temperature. An atomic force microscope (SPM-9600, SHIMADZU Co., Tokyo, Japan) was used to capture the surface change of cells on 20,000 magnification (at 1 Hz scan rate with tapping model and a spring constant of 10 N/m, and 3 × 3 μm scan range). *Ra* (Roughness average) was recorded to evaluate membrane surface roughness.

### Fluorescence Anisotropy Analysis

Fluorescence anisotropy analysis was performed according to the methods described by Meneghel et al. (2017) with slight modification. Briefly, cells with different treatments (BP, HP, HP-ES, and ES) were collected and washed twice with sterile water. Then cells were resuspended with PBS buffer (0.01 M, pH 7.0) containing 2 × 10^–6^ M 1,6-diphenyl-1,3,5-hexatriene (DPH), which was used as a probe to sense a change in membrane dynamics. Fluorescence anisotropy was determined by a fluorophotometer (EX: 360nm, EM: 430nm) with a polarization device. The fluorescence polarization (*p*) and anisotropy (*r*) were calculated as the followed equation:

$$p=\frac{I_{V\,V}-I_{V\,H}\,\left(I_{H\,V}/I_{H\,H}\right)}{I_{V\,V}+I_{V\,H}\,\left(I_{H\,V}/I_{H\,H}\right)}$$

$$r=\frac{2\,p}{3-p}$$

where *I* was fluorescence intensity and subscripts V (vertical) and H (horizontal) were considered as the orientation of polarizer and analyzer, respectively. All samples were carried out in three biological replications.

### Membrane Fatty Acids Analysis

The treated cells (BP, HP, HP-ES, and ES) were collected by centrifugation at 8000 × *g* for 5 min and washed twice with PBS buffer (0.01 M, pH 7.0). The extraction of membrane fatty acid methyl esters (FAMEs) was carried out by the method described in the literature (Zhao et al., 2021). The results were analyzed by GC-MS (Thermo trace1300-TSQ9000, NY, United States), and the FAMEs matched in the NIST05 library database, and the relative content of the FAMEs was calculated from peak areas. The ratio of unsaturated fatty acids to saturated fatty acids (U/S ratio) was calculated with the method described previously (Wu et al., 2012). All samples were carried out in three biological replications.

### Protein Preparation, TMT-Labeling and LC-MS/MS Analysis

Cells subjected to different treatments (BP, HP) were collected for protein extraction. Briefly, cells were transferred into 2 mL low protein binding tubes and lysed with 600 μL extraction buffer [Sucrose: 2.4 g, NaCl 0.058 g, EDTA⋅2Na 0.146 g, DTT 0.02 g, 0.5 M Tris-HCl (pH6.8) 2.5mL, 0.5 M Tris-HCl (pH8.8) 2.5mL, and add ddH_2_O to 10 mL] supplemented with PMSF to a final concentration of 1 mM. After lysis with sonication (80 W and 60 Hz for 3 min), samples were added with the same volume of Tris-phenol buffer (pH 7.8) and mixed for 30 min at 4°C. Then, the mixtures were centrifuged (8000 × *g* for 10 min at 4°C) to collect phenol supernatants. The supernatants were added with 5-fold volume of 0.1 M cold ammonium acetate-methanol buffer and precipitated at −20°C overnight. After precipitation completely, the samples were centrifuged (12000 × *g* for 10 min at 4°C) to collect precipitations. The precipitations were washed with cold methanol and then centrifuged (12000 × *g* for 10 min at 4°C) again to collect precipitations, repeated once. Then methanol was removed by washing twice with acetone. Then, the precipitations were collected with centrifugation at 12000 × *g* for 10 min at 4°C and dried at room temperature for 3 min, and then dissolved in lysis buffer (250 mM HEPES, 2% SDS, pH 7.0) for 3 h. Finally, the samples were centrifuged at 12000 × *g* for 10 min at room temperature to collect supernatants, repeated once. Protein concentration was determined by BCA assay (Thermo Scientific, United States).

Protein samples (50 μg) extracted from different groups of cell samples (BP, HP) were added with DTT to a final concentration of 5 mM and then incubated at 55°C for 30 min. Then iodoacetamide was added to a final concentration of 10 mM at room temperature in the dark for 15 min. Then the precooled acetone was used to precipitate the protein at −20°C overnight. After precipitation, the protein was collected by centrifugation (8000 × *g* for 10 min at 4°C). The enzymolysis diluent [protein:enzyme = 50:1 (m/m)] was added to redissolve the protein and then incubated for digestion with trypsin overnight at 37°C. Finally, samples were lyophilized after enzymolysis.

For TMT labeling, samples prepared above were resuspended in 50 μL 100 mM TEAB buffer (pH 8.0) in 1.5 ml tubes for labeling. Then 41 μL of TMT label reagent (Thermo Scientific, United States) was added to each sample for mixing at room temperature for 1 h. Finally, 8 μL of 5% hydroxylamine were added to terminate the reaction by incubation for 15 min.

Reversed-phase (RP) separation was performed on an 1100 HPLC System (Agilent). Mobile phases A (2% acetonitrile in HPLC water) and B (98% acetonitrile in HPLC water) were used for RP gradient. The solvent gradient was set as follows: 0∼8 min, 89% A; 8∼8.01 min, 98∼95% A; 8.01∼48 min, 95∼75% A; 48∼60 min, 75∼60% A; 60∼60.01 min, 60∼10% A; 60.01∼70 min, 10% A; 70∼70.01 min, 10∼98% A; and70.01∼75 min, 98% A. Tryptic peptides were separated at a fluent flow rate of 300 μL/min and monitored at 210 and 280 nm. Samples were collected for 8–60 min, and the eluent was collected in a centrifugal tube. Samples were recycled in this order until the end of the gradient. Subsequently, the lyophilized peptides were analyzed by tandem mass spectrometry (MS/MS) using Q-Exactive mass spectrometer (Thermo, United States) equipped with a Nanospray Flex source (Thermo, United States). Full MS scans were acquired in the mass range of 350–1500 m/z with a mass resolution of 60,000 and the AGC target value was set at 3e^6^. The 10 most intense peaks in MS were fragmented with higher-energy collisional dissociation (HCD) with an NCE of 32. MS/MS spectra were obtained with a resolution of 15,000 with an AGC target of 2e^5^ and a max injection time of 40 ms. The Q-E dynamic exclusion was set for 30.0 s and run under positive mode.

### Data Analysis and Bioinformatics

The mass spectrometry proteomics data have been deposited to the ProteomeXchange Consortium^1^ via the iProX partner repository with the dataset identifier PXD025119. The LC-MS/MS data were analyzed for protein identification and quantification using Proteome Discoverer 2.1 software (Thermo Fisher Scientific). The precursor MS 1 (mass tolerance in the first search) was set as 10 ppm, and MS 2 (mass tolerance in the second search) was set as 0.02 Da. In addition, FDR (false discovery rate) was adjusted to < 1%. Fold change (FC) ≥ 1.20 or ≤ 0.83-fold cut-off value was considered as DEP (differentially expressed protein) with a *p*-value of < 0.05. The Gene Ontology (GO) and Kyoto Encyclopedia of Genes and Genomes (KEGG) annotation and enrichment analysis were derived from the databases^2^ and^3^, respectively. The PPI (protein-protein interaction) networks and pathways were assessed by STRING database^4^.

### Statistical Analysis

Each experiment was carried out in three biological replications. Statistical differences are judged by One-way ANOVA with Duncan’s test with SPSS software (version 23.0 SPSS Inc., IBM company, New York, United States). Differences between groups with *p* < 0.05 (*n* = 3) are considered as statistically significant.

## Results

### Heat Preadaptation Improved the Resistance of *T. halophilus* to Multiple Stresses

**FIGURE 1:**
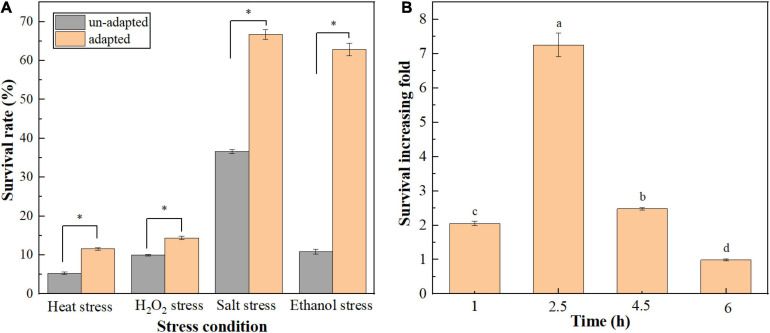
Effect of cross protection on the tolerance of *T. halophilus* to multiple environmental stresses. **(A)** Effect of heat preadaptation on the resistance to environmental stresses. Heat preadapted and un-adapted cells were treated by heat stress (60°C), H_2_O_2_ stress (0.03% H_2_O_2_), salt stress (35% NaCl), and ethanol stress (10% ethanol) for 2.5 h, respectively. **(B)** Effect of ethanol stress time on the survival increasing fold of *T. halophilus* during cross protection. Heat preadapted and un-adapted cells were treated by 10% ethanol stress for 1, 2.5, 4.5, and 6 h, respectively, and the survival rates were determined. *Error bars* indicate SD (*n* = 3). Significant differences among the groups are marked with “^∗^” or different letters (*p* < 0.05).

In the current study, the effect of heat preadaptation (45°C for 1.5 h) on the tolerance of *T. halophilus* to multiple stresses was investigated (Figure 1A). As shown in Figure 1A, heat preadaptation improved the resistance of cells to heat (60°C), oxygen (0.03% H_2_O_2_), salt (35% NaCl), and ethanol (10%) stresses, with 2. 18-, 1. 45-, 1. 83-, and 7.24-fold increase in survival rates, respectively. Those results suggested that cross protection could protect *T. halophilus* against, subsequently, stress conditions. Therefore, the effect of exposure time (10% ethanol for 0–6 h) on ethanol tolerance was investigated (Figure 1B). As shown in Figure 1B, higher survival of adapted cells was observed compared with cells subjected to ethanol stress directly (un-adapted cells) at various exposure times (1, 2.5, 4.5, and 6 h), and the survival increasing fold was the highest when cells were exposed at ethanol stress for 2.5 h. Therefore, the protective mechanisms of preadaptation were mainly focused on ethanol stress in this study.

### Changes in Cell Integrity Upon Exposure to Ethanol Stress

**FIGURE 2:**
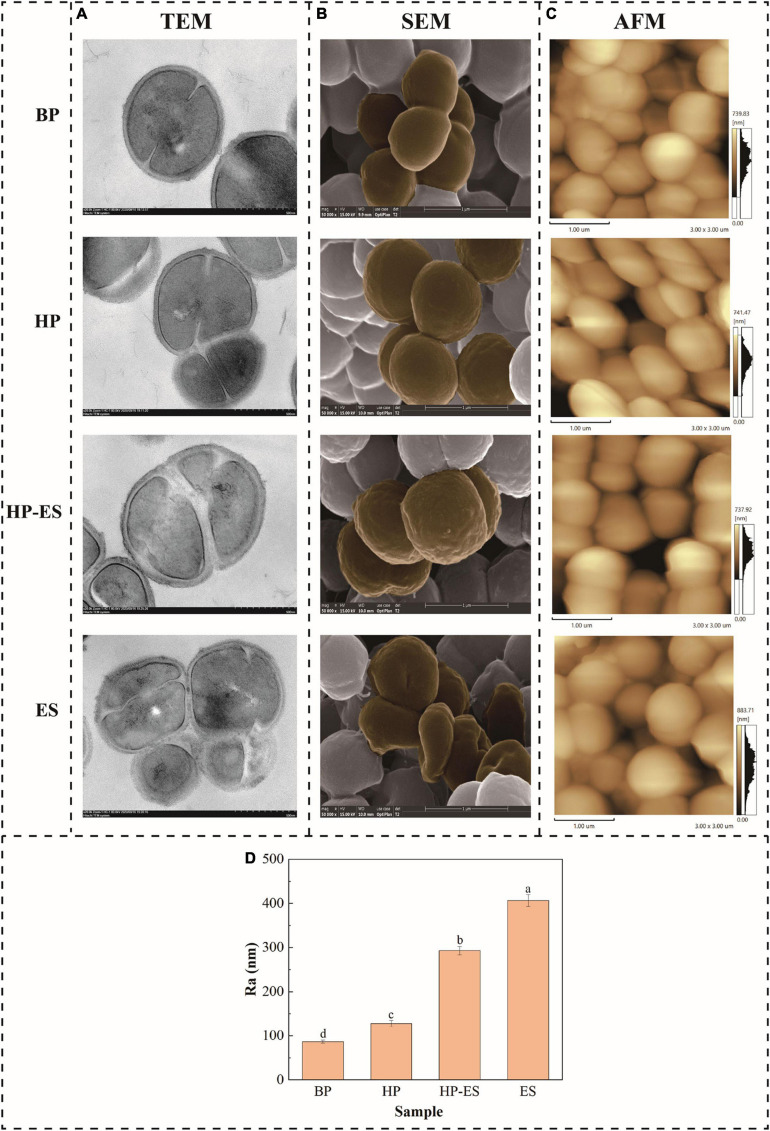
Changes in membrane integrity **(A)**, cell morphology **(B)**, and membrane roughness average **(C,D)** of *T. halophilus*. BP (before preadaptation): cells harvested at mid-exponential growth phase without any treatment; HP (heat preadaptation): cells were collected after heat preadaptation at 45°C for 1.5 h; HP-ES (heat preadaptation-ethanol stress): cells were heat preadapted at 45°C for 1.5 h and then challenged by 10% ethanol stress for 2.5 h; ES (ethanol stress): cells subjected to 10% ethanol stress for 2.5 h without heat preadaptation. The brown parts were added to show rough and broken parts. *Error bars* indicate SD (*n* = 3). Significant differences among the groups are marked with different letters (*p* < 0.05).

To further validate the effect of cross protection on the tolerance to ethanol stress, the cell membrane integrity of *T. halophilus* under ethanol stress was determined with TEM (Figure 2A). As shown in Figure 2A (TEM), cells in group BP and HP retained intact cell membranes, while cell membranes were cracked and their cytoplasm effused with cells exposed to ethanol stress. In the meanwhile, the degree of cracked cells was improved apparently in group HP-ES compared with ES cells. Those results mentioned above suggested that heat preadaptation may contribute to increased ethanol resistance by improving cell membrane integrity.

### Changes in Cell Surface Properties During Ethanol Stress

In this study, cell surface properties were evaluated via phenotype and membrane roughness by SEM and AFM analyses (Figures 2B,C). As the results of SEM shown in Figure 2B, the surface of BP cells was smooth and without a wrinkle. The shape of the HP cells changed slightly and kept a normal morphology. The morphology of ES cells was deformed severely and sunken wrinkles emerged on the surface of ES cells. However, preadaptation at 45°C for 1.5 h prior to ethanol stress (HP-ES) could significantly improve the morphology of the cell surface. Furthermore, the membrane roughness average (*Ra*) value of *T. halophilus* remarkably increased when cells encountered ethanol stress (ES and HP-ES) while BP and HP cells were at a low level (Figure 2D). Those results are consistent with SEM analysis and partially demonstrate that heat preadaptation contributes to the increasing stress tolerance of *T. halophilus* through maintaining a normal cell surface characterization.

### Changes in Membrane Fatty Acids After Ethanol Stress

**FIGURE 3:**
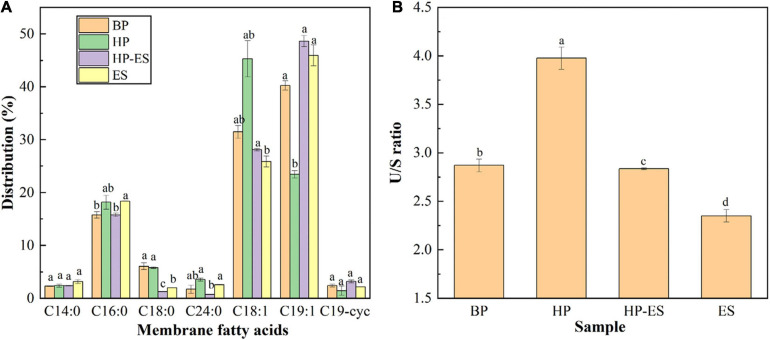
Analysis of membrane fatty acids in *T. halophilus* under ethanol stress. **(A)** the distribution of membrane fatty acids; **(B)** the ratio of un- to saturated fatty acids. BP (before preadaptation): cells harvested at mid-exponential growth phase without any treatment; HP (heat preadaptation): cells were collected after heat preadaptation at 45°C for 1.5 h; HP-ES (heat preadaptation-ethanol stress): cells were heat preadapted at 45°C for 1.5 h and then challenged by 10% ethanol stress for 2.5 h; ES (ethanol stress): cells subjected to 10% ethanol stress for 2.5 h without heat preadaptation. *Error bars* indicate SD (*n* = 3). Significant differences among the groups are marked with different letters (*p* < 0.05).

The distribution of membrane fatty acids in *T. halophilus* after different treatments (BP, HP, HP-ES, and ES) were determined in this study. As shown in Figure 3, the membrane fatty acids of *T. halophilus* mainly consisted of myristic acid (C_14:0_) palmitic acid (C_16:0_), stearic acid (C_18:0_), oleic acid (C_18:1_), nonadecylenic acid (C_19:1_), and cyclopropane fatty acid (C_19–cyc_). There was no significant change among those fatty acids between BP cells and HP cells or HP-ES cells and ES cells except C_18:1_ and C_19:1_. As for those two unsaturated fatty acids, the content of C_18:1_ in group HP was higher than BP while inversed in C_19:1_. When we analyzed the ratio of unsaturated to saturated fatty acid (U/S), we found the U/S ratio of HP cells was the highest (Figure 3B). In addition, cells challenged with ethanol stress directly (ES) showed a lower U/S than HP-ES cells. Those results suggested that cross protection improved the ethanol stress of *T. halophilus* by regulation of the distribution of membrane fatty acids and U/S ratio.

### Changes in Membrane Fluidity Under Ethanol Stress

**FIGURE 4:**
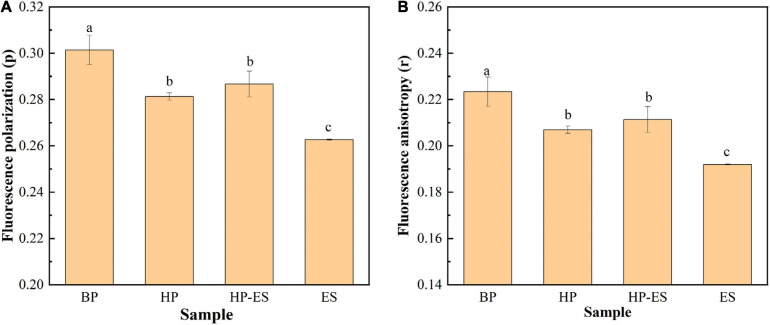
Changes in membrane fluidity of *T. halophilus* under ethanol stress. **(A)** fluorescence polarization (*p*); **(B)** fluorescence anisotropy (*r*). BP (before preadaptation): cells harvested at mid-exponential growth phase without any treatment; HP (heat preadaptation): cells harvested at mid-exponential growth phase were preadapted at 45°C for 1.5 h; HP-ES (heat preadaptation-ethanol stress): cells were preadapted at 45°C for 1.5 h and then subjected to 10% ethanol stress for 2.5 h; ES (ethanol stress): cells subjected to 10% ethanol stress for 2.5 h without heat preadaptation. *Error bars* indicate SD (*n* = 3). Significant differences among the groups are marked with different letters (*p* < 0.05).

The membrane fluidity of *T. halophilus* was determined by DPH probe, and the fluorescence polarization (*p*) and anisotropy (*r*) were monitored to evaluate the fluidity of the cell membrane. Figure 4A showed that fluorescence polarization decreased when cells were subjected to ethanol stress and HP-ES cells exhibited higher polarization than ES cells, indicating that ethanol may be a factor that affected the viscosity of the cell membrane. In addition, Figure 4B showed the change of fluorescence anisotropy in the cell membrane, and the results demonstrated that fluorescence anisotropy in BP cells was the highest among the four different treatments while the same with ES cells was the lowest, which showed cell membrane fluidity increased after ethanol stress. Those results suggested that ethanol stress led to the decrease of fluorescence anisotropy and the increase of membrane fluidity and heat preadaptation may help *T. halophilus* keep the original status as BP cells.

### Overview of Proteomic Analysis

**FIGURE 5:**
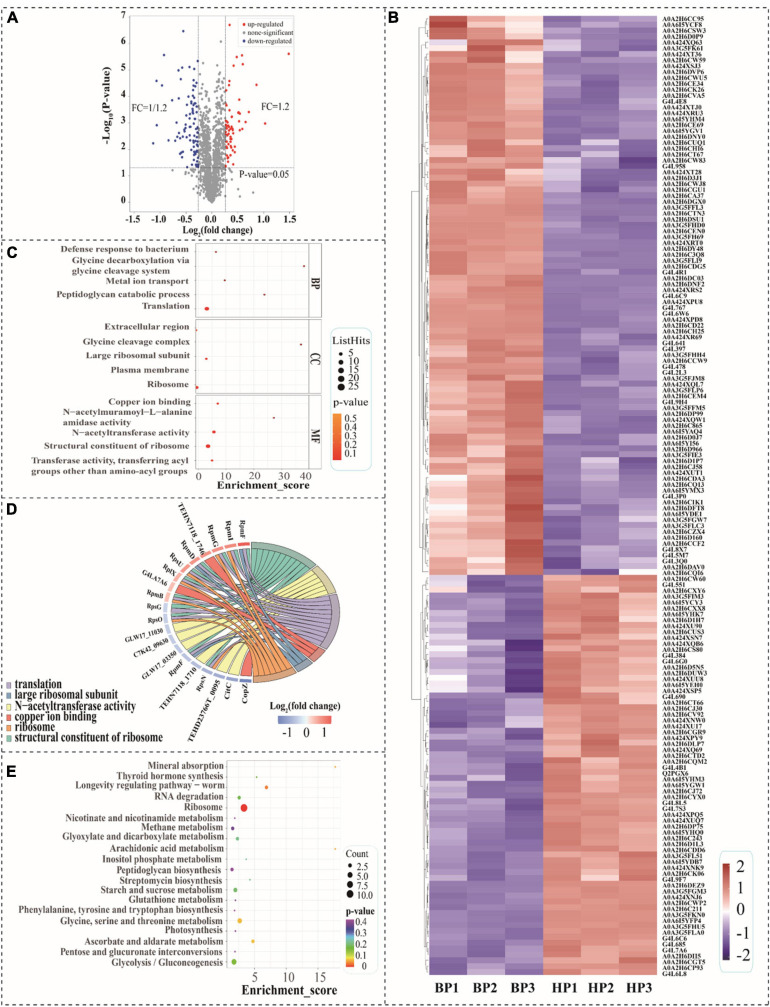
Analysis of differentially expressed proteins (DEPs) at proteomic level. **(A)** Volcano diagram of the proteins after heat preadaptation; **(B)** Heat map of DEPs after heat preadaptation, BP1–BP3: before preadaptation, HP1–HP3: heat preadaptation; **(C)** The top 5 GO terms of three gene ontology categories, BP, biological process; CC, cellular component; MF, molecular function; **(D)** Chord chart for the DEPs after heat preadaptation; and **(E)** KEGG enrichment analysis of the top 20 pathways.

In this study, TMT-based proteomic technology was employed to reveal the mechanisms of cross protection induced by heat preadaptation in *T. halophilus*. Proteins in cells with/without heat preadaptation (HP: heat preadaptation; BP: without heat preadaptation) were extracted for proteomic analysis. The result of the correlation test was shown in Supplementary Figure 1, which suggested that the data in this study was reliable. In addition, proteins with expression level ≥ 1.20 or ≤ 0.83-fold and *p*-value < 0.05 were considered as differentially expressed proteins (DEPs). Figure 5 demonstrated the DEPs at the proteomic level. As showed in Figures 5A,B, a total of 1940 proteins were identified and 163 proteins were differentially expressed including 68 proteins up-regulated and 95 proteins down-regulated (Figures 5A,B and Supplementary Table 1).

Gene ontology and KEGG functional enrichment analysis of DEPs were carried out in this study (Figures 5C–E). Figure 5C showed the top 5 terms of each category which were classified in biological process (BP), cellular component (CC), and molecular function (MF). As for biological process (BP), the DEPs were enriched in the “translation,” “peptidoglycan catabolic process,” “metal ion transport,” “glycine decarboxylation via glycine cleavage system” and “defense response to bacterium.” Within the cellular component (CC) category, the top five terms of frequency were “ribosome,” “plasma membrane,” “large ribosomal subunit,” “glycine cleavage complex” and “extracellular region.” In the molecular function (MF), the largest five subcategories of DEPs belonged to “structural constituent of ribosome,” “transferase activity, transferring acyl groups other than amino-acyl groups,” “N-acetyltransferase activity,” “N-acetylmuramoyl-L-alanine amidase activity,” and “copper ion binding.” As shown in Figure 5D, the chord chart showed the DEPs enriched in more than three subcategories during GO enrichment analysis, which were “structural constituent of ribosome,” “N-acetyltransferase activity,” “translation,” “copper ion binding,” “large ribosomal subunit” and “ribosome.” Those results suggested that genetic information processing was tightly associated with the response mechanism of heat preadaptation.

In order to investigate the biological pathways affected by heat preadaptation, we mapped the DEPs in 43 pathways (Supplementary Figure 2), and the top 20 of those KEGG pathways were displayed in Figure 5E. The most abundant categories (enriched in more than three pathways) on the KEGG classification level were “amino acid metabolism, 3,” “carbohydrate metabolism, 10,” “energy metabolism, 3,” “lipid metabolism, 3,” “membrane transport, 3” and “genetic information processing, 6,” which indicated that *T. halophilus* may regulate these metabolic pathways in response to heat preadaptation. Therefore, the DEPs involved in these categories were discussed to further reveal the potential mechanisms of heat preadaptation.

### DEPs Involved in Heat Preadaptation

**FIGURE 6:**
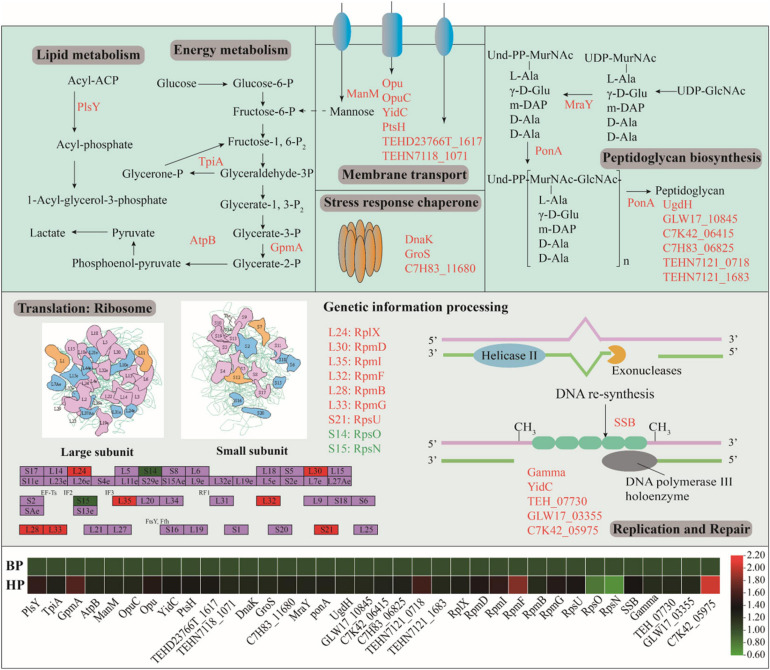
Effect of heat preadaptation on protein expression involved in cellular metabolism, membrane transport, peptidoglycan biosynthesis and genetic information process. The up- and down-regulated proteins after heat preadaptation are shown in red and green, respectively. The changes of proteins in the pathway are shown in the heat map. BP (before preadaptation): cells harvested at mid-exponential growth phase without any treatment; HP (heat preadaptation): cells harvested at mid-exponential growth phase were preadapted at 45°C for 1.5 h.

Figure 6 showed the DEPs involved in cellular metabolisms after heat preadaptation. In detail, heat preadaptation led to differential expression of proteins involved in energy metabolism including triosephosphate isomerase (TpiA), putative hydrolase (GpmA), and ATP synthase subunit a (AtpB), and 1. 28-, 1. 64-, and 1.22-fold up-regulation were observed, respectively. As for membrane transport, 7 up-regulated DEPs were mannose/glucose-specific phosphotransferase system enzyme IIC component (ManM), osmoprotectant ABC transporter substrate-binding protein (OpuC), putative osmoprotectant ABC transporter permease/substrate-binding protein (Opu), membrane protein insertase YidC (YidC), phosphocarrier protein HPr (PtsH), putative ABC transporter permease protein (TEHD23766T_1617) and putative small-conductance mechanosensitive channel (TEHN7118_1071). Additionally, eight DEPs: “phospho-N-acetylmuramoyl-pentapeptide-transferase, MraY,” “DD-transpeptidase, PonA,” “UDP-glucose 6-dehydrogenase, UgdH,” “LysM peptidoglycan-binding domain-containing protein, GLW17_10845,” “glycoside hydrolase family 25, C7K42_06415,” “N-acetylmuramoyl-L-alanine amidase, C7H83_06825,” “putative LysR family transcriptional regulator, TEHN7121_0718,” and “putative N-acetylmuramoyl-L-alanine amidase, TEHN7121_1683,” which was involved in peptidoglycan biosynthesis, were significantly up-regulated during heat adaptation. Meanwhile, proteins involved in lipid metabolism (glycerol-3-phosphate acyltransferase, PlsY), signal conduction [probable 2-(5″-triphosphoribosyl)-3′-dephosphocoenzyme-A synthase, CitG], and classic stress response chaperones (chaperone protein DnaK, DnaK; 10 kDa chaperonin, GroS; universal stress protein, C7H83_11680) were also differentially expressed after heat preadaptation at 45°C for 1.5 h.

Furthermore, we analyzed the DEPs mapped in the KEGG pathways and found proteins involved in “genetic information processing” significantly expressed after heat preadaptation. As shown in Figure 6, nine proteins involved in the “ribosome” were overexpressed in response to heat preadaptation (50S ribosomal protein L24, RplX; “50S ribosomal protein L30, RpmD; 50S ribosomal protein L35, RpmI; 50S ribosomal protein L38, RpmF; 50S ribosomal protein L28, RpmB; 50S ribosomal protein L33, RpmG; 50S ribosomal protein S21, RpsU; 30S ribosomal protein S15, RpsO; 30S ribosomal protein S14, RpsN). As for “replication and repair,” “DNA replication” (single-stranded DNA-binding protein, SSB; metalloregulator ArsR/SmtB family transcription factor, GLW17_03355; TetR/AcrR family transcriptional regulator, C7K42_05975“), “RNA degradation” (chaperone protein DnaK, DnaK), “cell cycle” (cell division protein DivIB, FtsQ), and “protein export” (membrane protein insertase YidC, YidC) pathways were also be activated after heat preadaptation.

### Protein-Protein Interaction (PPI) Network Analysis

**FIGURE 7:**
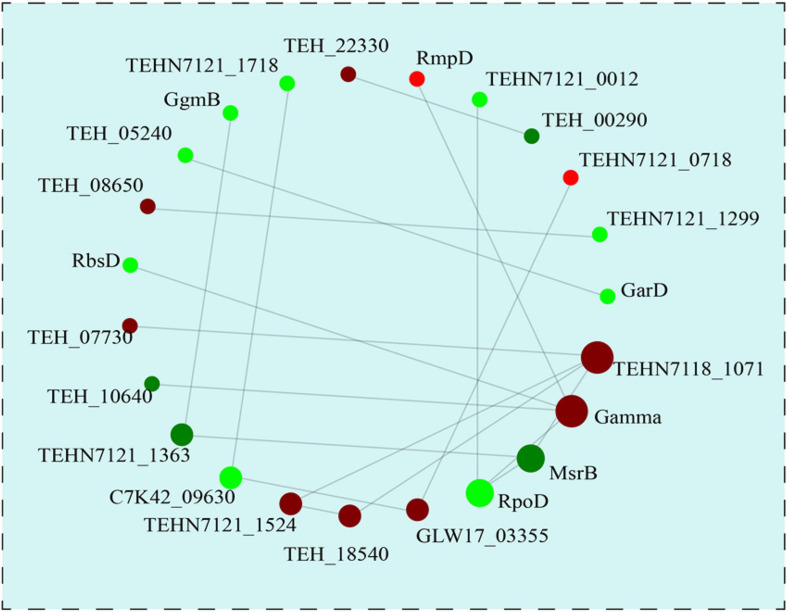
The protein-protein interaction network analysis of the differentially expressed proteins. With the fold change >1.2 or <0.83, all the 23 differentially DEPs were used for PPI analysis. The up- and down-regulated proteins after heat preadaptation are shown in red and green, respectively.

In order to understand the underlying protein-protein interaction (PPI) of these DEPs, PPI network analysis was subsequently performed by using STRING software (Figure 7). A total of 23 DEPs were identified in this interaction network and the most interactive proteins were involved in membrane transport (TEHN7118_1071, putative small-conductance mechanosensitive channel), genetic information processing (Gamma, DNA topoisomerase; rpoD, RNA polymerase sigma factor SigA), and stress response (msrB, Peptide methionine sulfoxide reductase MsrB), which suggested the potential roles of these proteins during heat preadaptation in *T. halophilus*.

## Discussion

Cross protection is a universally utilized mechanism used by eukaryote and prokaryote, which confer cells higher resistance during harsh stress conditions by preadaptation at mild stress conditions. In this study, cross protection was successfully employed by *T. halophilus* to improve the stress resistance to heat, oxygen, and salt (2. 18-, 1. 45-, and 1.83-fold increase in survival) of cells, especially for ethanol stress (7.24-fold increase in survival under 10% ethanol for 2.5 h) (Figure 1). Similar results preformed in *A. acidoterrestris* and *Salmonella enterica* were also reported by the researchers that an increase in survival under thermal stress (65°C, 5 min) from 14.72 to 62.09% in *A. acidoterrestris* or an improvement of ethanol tolerance (26.39-fold increase in survival under 15% ethanol, 1 h) for *S. enterica* was observed through cross protection (He et al., 2019; Zhao et al., 2021). These results indicated that heat preadaptation may be considered as a potential strategy to engineer the robustness of strains used during food fermentation.

Then, the protective mechanisms of cross protection were elucidated by focusing on physiological properties and proteomic analysis. As is known, ethanol is a toxic factor that could disturb the metabolism and injure the plasma membrane of cells (Wijesundara and Rupasinghe, 2019). Therefore, the integrity of cell membrane evaluated by TEM analysis was performed to investigate how heat preadaptation contributed to improving the ethanol resistance of *T. halophilus* in this study (Figure 2A). As shown in Figure 2A (TEM), ethanol severely damaged cell membrane of *T. halophilus*, which resulted in a low survival after ethanol stress (ES cells in Figure 1). While cells treated by heat preadaptation before ethanol stress (HP-ES), higher integrity was maintained than that of ES cells, which was consistent with the results showed in Figure 1. Therefore, we infer that maintenance of membrane integrity may be one of the protective mechanisms of cross protection in *T. halophilus*.

In addition, the morphology of cells was considered as an indicator to evaluate the growth condition or tolerance of cells under environmental stresses (Yang et al., 2018; Wang et al., 2020). In the present study, the phenotype observed by SEM (Figure 2B) demonstrated that ethanol stress resulted in the deformation of *T. halophilus* cells (HP-ES and ES in Figure 2B). A similar result also reported that ethanol destroyed the cell membrane and affected the industrial performance of *O. oeni* (Bonomo et al., 2018). In this study, the degree of cell deformation was apparently improved after preadaptation at 45°C for 1.5 h (Figure 2B, HP-ES), which suggested that heat preadaptation contributed to ethanol resistance by maintaining the morphology of cells at a normal condition. Meanwhile, the change of membrane roughness was considered as an indicator of the growth condition in microorganisms. Figure 2D showed that the roughness average of HP-ES cells significantly decreased compared to ES cells, which indicated that cells kept a smooth surface after heat preadaptation. Those results were consistent with the report by Wang et al. (2020) that heat preadaptation contributed to improving the salt tolerance of *Zygosaccharomyces rouxii* by regulating the membrane roughness of cells.

Generally, the cytoplasmic membrane is considered as the first boundary for microorganisms to contact the environmental stress conditions. Therefore, the functional properties of membrane, including the distributions of membrane fatty acids, membrane fluidity, and membrane integrity, play vital roles in protecting cells against stress conditions (Wu et al., 2012; Tang et al., 2014; Huang et al., 2016). In this study, the categories and relative amount of membrane fatty acids of *T. halophilus* were calculated after GC-MS analysis. The contents of oleic acid (C_18:1_), reported as a vital unsaturated fatty acid to defense various stress conditions, increased in the HP cells compared to BP cells, which may contribute to improving the survival rate of *T. halophilus* under the subsequent ethanol stress. Similar work was also reported by Huang et al. (2016) that acid preadaptation at pH 4.5 for 2 h induced cross protection to help *Lactobacillus plantarum* against subsequent acid stress (pH 3.0 for 1 h) with an up-regulation of oleic acid (C_18:1_) abundance. Besides, regulation of the U/S ratio was considered as a common strategy for microbial cells to cope with stress conditions (Shin et al., 2018). As shown in Figure 3B, the result demonstrated that ethanol stress led to a decrease in U/S ratio (ES cells), and this tendency could be alleviated by heat preadaptation, which indicated that heat preadaptation may benefit to help cells cope with ethanol by maintaining U/S ratio at a higher level. In addition to the distribution of membrane fatty acid, membrane fluidity was also regarded as another indicator to evaluate the state of the cytoplasmic membrane under ethanol stress (Margalef-Catala et al., 2016). In this study, membrane fluidity was determined by the assay of fluorescence anisotropy with a DPH probe (Figure 4). In detail, the ES cells exhibited the lowest value of anisotropy suggesting the highest fluidity among the four groups. Generally, the increase of fluidity may result in the entrance of toxic matrixes (ethanol mainly in this study) into the cells, and, subsequently, disturb the cellular metabolisms, injure the membrane structure or even kill cells, which partially explained the results shown in Figure 1. Additionally, previous research also showed that ethanol stress led to a significant increase of membrane fluidity in *O. oeni* (Margalef-Catala et al., 2016). Those results revealed that heat preadaptation was beneficial to maintain the fluidity at a normal level (HP-ES cells in Figure 4B) and may contribute to help cells adapt to ethanol stress.

In order to further uncover the protective mechanisms of cross protection comprehensively, proteomic strategy was utilized in the current study. Previous research indicated that lactic acid bacteria generally strengthened their cellular metabolisms in response to harsh conditions (Wu et al., 2018; Zhu et al., 2019; Zhai et al., 2020). In this work, the proteomic results showed that 163 proteins were differentially expressed after heat preadaptation, and energy metabolism, membrane transport, peptidoglycan biosynthesis, and ribosomes were the most abundant metabolic pathways based on GO and KEGG enrichment analyses. Energy metabolism, an essential process, plays a vital role in the growth of microorganisms. As shown in Figure 6, GpmA, AtpA, and TpiA were all up-regulated in response to heat preadaptation. GpmA (putative hydrolase) and AtpB (ATP synthase subunit a) were associated with the glycolysis pathway and oxidative phosphorylation (Radin et al., 2019; Dominguez-Ramirez et al., 2020). TpiA (triosephosphate isomerase) may contribute to the uptake of extracellular matrixes including aminoglycoside and activate the process of energy production in oxidative phosphorylation, carbon metabolism, and respiration (Chen et al., 2017; Xia et al., 2020). In addition, McCloskey et al. (2018) reported that deletion of *tpiA* led to a negative effect on the growth rate and glycolysis pathway in *E. coli*. Therefore, the upregulation of these proteins may contribute to produce higher amounts of ATP and improve the resistance of *T. halophilus* under the subsequent ethanol stress.

Membrane transport is commonly employed by microorganisms to uptake extracellular organic compounds under different growth conditions. Membrane protein insertase (YidC) was significantly up-regulated after heat preadaptation in this study which may benefit the uptake of extracellular nutrients. Kuhn and Kiefer (Kuhn and Kiefer, 2017) suggested that YidC could promote the lateral movement of transmembrane domains of membrane proteins into the lipid bilayer during protein biogenesis, which indicated that YidC may be one of the underlying response mechanisms of *T. halophilus* during heat preadaptation. In addition, phosphocarrier protein HPr in the phosphotransferase system (PTS) was also significantly up-regulated in response to heat preadaptation, which may promote the uptake of nutrients and contribute to enhancing the stress resistance of *T. halophilus* under ethanol stress. Previous research showed that HPr may contribute to improve the resistance and adaptation to various stress conditions. For example, He et al. (2016a) reported that *T. halophilus* induced the expression of HPr during acid adaptation and increased the resistance to the following lethal acidic stress encountered. In addition, Gao et al. (2019) also reported that HPr dramatically affected the resistance to oxidative stress, matrixes uptake, and biofilm formation in *B. cereus* 905. Furthermore, the results of PPI network analysis showed that protein (TEHN7118_1071, putative small-conductance mechanosensitive channel) involved in membrane transport had the most interactive, which indicated that membrane transport may be a vital metabolic pathway for *T. halophilus* in response to heat preadaptation (Figure 7). Overall, these results demonstrated that heat preadaptation improved the stress resistance of *T. halophilus* by activating the proteins participated in membrane transport.

Cell membrane and cell wall, considered as the cell barriers of microorganisms, were reported to protect the cell from environmental stresses. Phospholipids are an indispensable component of cell membranes and PlsY (glycerol-3-P transferase) is the first step in the biosynthesis of membrane phospholipid, which played a critical role for cell defense against environmental stresses (Cherian et al., 2012; Li et al., 2017). In this study, PlsY was up-regulated 1.5 times after heat preadaptation, which may strengthen the process of lipid biosynthesis and contribute to improving the tolerance of cells to ethanol stress. In addition, peptidoglycan biosynthesis is considered a common strategy for bacteria to cope with various stress conditions (He et al., 2016a; Liu et al., 2018; Hengel et al., 2020). In this study, proteins MraY (phospho-N-acetylmuramoyl-pentapeptide-transferase), PonA (DD-transpeptidase), and UgdH (UDP-glucose 6-dehydrogenase) involved in peptidoglycan biosynthesis were also up-regulated after heat preadaptation, which suggested that peptidoglycan biosynthesis may be related to heat preadaptation and contribute to helping *T. halophilus* adapt to the ethanol condition.

Additionally, a total of 3 proteins (DnaK, GroS, and C7H83_11680) affiliated to stress response chaperones were identified and the expressions were up-regulated. Generally, stress response chaperones showed a positive effect on the resistance of cells to various stresses condition. It was reported that the overexpression of DnaK and GroEL helped *T. halophilus* and *Lactobacillus sanfranciscensis* combat acid stress and high-pressure stress, respectively (Hormann et al., 2006; He et al., 2016a; Chen et al., 2017). In addition, DnaK could restrain protein aggregation and contribute to improving the resistance to thermal stress in *Lactococcus lactis* NZ9000 (Abdullah Al et al., 2010). In this work, the up-regulation of these molecular chaperones in response to heat preadaptation may contribute to improving the resistance to ethanol stress of *T. halophilus*.

Genetic information processing was essential to the growth and proliferation of lactic acid bacteria. For instance, the process of translation and DNA replication were indispensable for microbes to transmit genomic information (He et al., 2019; Yao et al., 2019). He et al. (2019) reported that the upregulation of ribosome-related proteins contributed to improving the stress resistance of *S. enterica* under high ethanol conditions. In this manuscript, the expressions of 7 ribosomal proteins (RplX, RpmD, RpmI, RpmF, RpmB, RpmG, and RpsU) were remarkably increased in response to heat preadaptation (Figure 6). A similar result reported that preadaptation at a mild stress condition (heat, cold, acid, or bile salt) led to the up-regulation of 30S ribosomal protein 21 (RpsU) in *Lactobacillus kefiranofaciens*, which was beneficial to help *L. kefiranofaciens* to cope with various environmental stresses (Chen et al., 2017). Besides, 50S ribosomal protein (Rpm) was reported to improve acid resistance of *Lactobacillus pentosus* (Perez Montoro et al., 2018). These results suggested that upregulation of ribosomal proteins may contribute to help *T. halophilus* deal with stressful conditions. In addition, SSB (single-stranded DNA-binding protein) in *T. halophilus* was also upregulated when cells were subjected to heat preadaptation at 45°C for 1.5 h. Previous research reported that overexpression of SSB from *Xanthomonas oryzae* significantly enhanced resistance of *Nicotiana benthamiana* to salt stress (Cao et al., 2018). Additionally, up-regulation of SSB was also detected in *T. halophilus* during acid preadaptation reported by He et al. (2016a). These results verified that SSB may be a key and potential protein to protect cells against multiple stress conditions. It was also worth noting that the expression of cell division protein DivIB (FtsQ) was significantly increased during heat preadaptation in this study. Generally, cell division would be activated during cell growth especially under stress condition (Sureka et al., 2010). Therefore, the activation of proteins involved in genetic information processing may contribute to the survival of cells during environmental stresses.

## Conclusion

In this study, cross protection induced by heat preadaptation was successfully applied in *T. halophilus* to improve the resistance to multiple environmental stresses, especially to ethanol stress. In order to explore the protective mechanisms of cross protection in *T. halophilus*, cell physiological properties and TMT-labeled proteomic analysis were performed. The results suggested that cross protection induced by heat preadaptation protected cells against ethanol stress by maintaining cell surface properties and regulating the membrane fatty acids composition and membrane fluidity. Proteomic analysis showed that 163 proteins were differentially expressed after heat preadaptation at 45°C for 1.5 h and the functions of DEPs were also discussed in this study. Therefore, this study may be beneficial to understanding the protective mechanisms of temperature-induced cross protection and contributing to enhancing the stress tolerance of cells during food fermentation.

## Data Availability Statement

The original contributions presented in the study are publicly available. This data can be found here: the ProteomeXchange Consortium (http://proteomecentral.proteomexchange.org) via the iProX partner repository with the dataset identifier PXD025119.

## Author Contributions

HY carried out all experimental work, wrote the original draft, and revised the manuscript. SY and MZ contributed to the design and analyzed data. CW edited the manuscript and supervised the study. All authors approved the final version of the manuscript and agreed to be accountable for all aspects of the work.

## Conflict of Interest

The authors declare that the research was conducted in the absence of any commercial or financial relationships that could be construed as a potential conflict of interest.

## Footnotes

1 http://proteomecentral.proteomexchange.org

2 http://www.ebi.ac.uk/GOA/

3 https://www.kegg.jp/kegg/

4 http://string-db.org
