# Supplementary material for: Heat Adaptation Induced Cross Protection Against Ethanol Stress in Tetragenococcus halophilus: Physiological Characteristics and Proteomic Analysis
Source: Front Microbiol. 2021 Jun 18;12:686672. doi: 10.3389/fmicb.2021.686672 (PMC8249775; doi:10.3389/fmicb.2021.686672)
Supplement: Supplementary file 1 [file Data_Sheet_1.docx]

**TABLE | S1** The profile of DEPs of *T. halophilus* after heat preadaptation.

**FIGURE | S1** The quality analysis of proteome after heat preadaptation. A: correlation analysis of samples; B: trusted proteins analysis with box plots.

**FIGURE | S2** KEGG analysis of DEPs in *T. halophilus* after heat preadaptation. A: the categories of KEGG functional enrichment analysis at level 1; B: the categories of KEGG functional enrichment analysis at level 2.

**Table S1** the profile of DEPs of *T. halophilus* after heat preadaptation.

| **Accession** | **Gene Name** | **Description** | **P-value** | **Fold Change** |
| --- | --- | --- | --- | --- |
| Up-regulation | | | | |
| A0A424XSN7 | *groS* | 10 kDa chaperonin | 0.0172 | 1.22 |
| A0A3G5FKN0 | *rpsU* | 30S ribosomal protein S21 | 0.0000 | 1.39 |
| A0A2H6CDD6 | *rplX* | 50S ribosomal protein L24 | 0.0003 | 1.27 |
| A0A2H6CK06 | *rpmB* | 50S ribosomal protein L28 | 0.0022 | 1.20 |
| A0A424XPQ5 | *rpmD* | 50S ribosomal protein L30 | 0.0004 | 1.42 |
| A0A424XNJ6 | *rpmF* | 50S ribosomal protein L32 | 0.0000 | 1.82 |
| A0A424XUQ7 | *rpmG* | 50S ribosomal protein L33 | 0.0001 | 1.44 |
| A0A3G5FGM3 | *rpmI* | 50S ribosomal protein L35 | 0.0000 | 1.50 |
| A0A6I5YEH0 | *argS* | Arginine--tRNA ligase | 0.0216 | 1.23 |
| A0A424XU17 | *atpB* | ATP synthase subunit a | 0.0051 | 1.22 |
| A0A2H6CQM2 | *tpiA* | Triosephosphate isomerase | 0.0034 | 1.28 |
| A0A6I5YHM3 | *ugdH* | UDP-glucose 6-dehydrogenase | 0.0079 | 1.21 |
| A0A2H6CXX8 | *Opu* | Putative osmoprotectant ABC transporter permease/substrate-binding protein | 0.0019 | 1.45 |
| A0A2H6D5N5 | *ftsQ* | Cell division protein DivIB | 0.0052 | 1.28 |
| A0A6I5YHK7 | *dnaK* | Chaperone protein DnaK | 0.0089 | 1.23 |
| G4L7A6 | *copZ* | Copper chaperone CopZ | 0.0008 | 1.21 |
| A0A3G5FL51 | *C7H83_11750* | Damage-inducible protein CinA | 0.0013 | 1.25 |
| A0A2H6CTD2 | *ponA* | DD-transpeptidase | 0.0014 | 1.26 |
| A0A2H6CXY6 | *gamma* | DNA topoisomerase | 0.0422 | 1.20 |
| G4L551 | *TEH_03480* | DUF1858 domain-containing protein | 0.0085 | 1.21 |
| A0A2H6CJ72 | *TEHD23766T_2463* | DUF4352 domain-containing protein | 0.0012 | 1.21 |
| A0A424XPY9 | *C7K42_05700* | DUF979 domain-containing protein | 0.0064 | 1.25 |
| A0A2H6CV92 | *prsA* | Foldase protein PrsA | 0.0006 | 1.31 |
| A0A424XNW0 | *C7K42_10255* | Glutaconyl-CoA decarboxylase subunit beta | 0.0041 | 1.30 |
| G4L8L5 | *plsY* | Glycerol-3-phosphate acyltransferase | 0.0017 | 1.50 |
| A0A424XQB6 | *C7K42_06415* | Glycoside hydrolase family 25 | 0.0204 | 1.25 |
| A0A6I5YDB7 | *GLW17_04960* | Glycosyltransferase | 0.0004 | 1.45 |
| A0A2H6CGR9 | *TEHD23766T_1620* | HicB-like domain-containing protein | 0.0010 | 1.23 |
| A0A3G5FHU5 | *C7H83_05215* | IS30 family transposase | 0.0000 | 2.79 |
| A0A2H6C243 | *TEHN7118_1071* | Putative small-conductance mechanosensitive channel | 0.0013 | 1.22 |
| A0A2H6D1H7 | *Ssb* | Single-stranded DNA-binding protein | 0.0129 | 1.36 |
| A0A424XUU8 | *C7K42_03280* | CDP-glycerol glycerophosphotransferase family protein | 0.0371 | 1.33 |
| A0A6I5YHQ0 | *GLW17_10845* | LysM peptidoglycan-binding domain-containing protein | 0.0016 | 1.21 |
| G4L685 | *manM* | Mannose/glucose-specific phosphotransferase system enzyme IIC component | 0.0002 | 1.22 |
| A0A6I5YFP4 | *yidC* | Membrane protein insertase YidC | 0.0000 | 1.34 |
| A0A6I5YGW1 | *GLW17_03355* | Metalloregulator ArsR/SmtB family transcription factor | 0.0028 | 1.22 |
| A0A3G5FIM3 | *C7H83_06825* | N-acetylmuramoyl-L-alanine amidase | 0.0017 | 1.28 |
| A0A6I5YCY3 | *opuC* | Osmoprotectant ABC transporter substrate-binding protein | 0.0016 | 1.22 |
| Q2PGX6 | *ptsH* | Phosphocarrier protein HPr | 0.0019 | 1.32 |
| A0A2H6DLP7 | *mraY* | Phospho-N-acetylmuramoyl-pentapeptide-transferase | 0.0026 | 1.25 |
| A0A424XNK9 | *citG* | Probable 2-(5''-triphosphoribosyl)-3'-dephosphocoenzyme-A synthase OS | 0.0002 | 1.20 |
| A0A424XSP5 | *dapE* | Probable succinyl-diaminopimelate desuccinylase | 0.0437 | 1.20 |
| A0A2H6CGT5 | *TEHD23766T_1617* | Putative ABC transporter permease protein | 0.0017 | 1.32 |
| A0A2H6CJ30 | *gpmA* | Putative hydrolase | 0.0004 | 1.64 |
| G4L6C6 | *TEH_07730* | Putative ribonuclease | 0.0001 | 1.21 |
| A0A424XQ69 | *C7K42_05975* | TetR/AcrR family transcriptional regulator | 0.0011 | 2.04 |
| A0A2H6DUW3 | *Trx* | Thioredoxin | 0.0168 | 1.28 |
| A0A2H6CYX0 | *TEHN7121_0718* | Putative LysR family transcriptional regulator | 0.0009 | 1.57 |
| A0A2H6CP93 | *TEHN7118_1746* | Putative metal-binding protein | 0.0004 | 1.43 |
| A0A2H6D1L3 | *TEHN7121_1683* | Putative N-acetylmuramoyl-L-alanine amidase | 0.0009 | 1.22 |
| A0A2H6DEZ9 | *TEHN7121_1524* | Uncharacterized protein | 0.0000 | 1.27 |
| A0A424XU90 | *C7K42_03935* | Uncharacterized protein | 0.0041 | 1.28 |
| G4L384 | *TEH_22330* | Uncharacterized protein | 0.0076 | 1.29 |
| A0A2H6CUS3 | *TEHN7118_1551* | Uncharacterized protein | 0.0025 | 1.33 |
| G4L690 | *TEH_07370* | Uncharacterized protein | 0.0028 | 1.20 |
| A0A2H6CS80 | *TEHN7118_0656* | Uncharacterized protein | 0.0294 | 1.24 |
| A0A2H6CT66 | *TEHN7118_0991* | Uncharacterized protein | 0.0049 | 1.20 |
| A0A2H6CW60 | *TEHN7118_2034* | Uncharacterized protein | 0.0079 | 1.36 |
| A0A2H6CWP2 | *TEHN7118_2215* | Uncharacterized protein | 0.0000 | 1.51 |
| G4L4B1 | *TEH_24630* | Uncharacterized protein | 0.0030 | 1.23 |
| G4L6L8 | *TEH_08650* | Uncharacterized protein | 0.0014 | 1.30 |
| G4L7S3 | *TEH_12700* | Uncharacterized protein | 0.0019 | 1.26 |
| G4L9F7 | *TEH_18540* | Uncharacterized protein | 0.0086 | 1.30 |
| A0A3G5FLA0 | *C7H83_11680* | Universal stress protein | 0.0000 | 1.26 |
| Down-regulation | | | | |
| A0A3G5FLP6 | *citC* | [Citrate [pro-3S]-lyase] ligase | 0.0034 | 0.63 |
| A0A424XQL7 | *rpsN* | 30S ribosomal protein S14 | 0.0021 | 0.69 |
| A0A424XPD8 | *rpsO* | 30S ribosomal protein S15 | 0.0000 | 0.81 |
| A0A3G5FFL3 | *rpsG* | 30S ribosomal protein S7 | 0.0000 | 0.83 |
| A0A3G5FGW7 | *rpmF* | 50S ribosomal protein L32 | 0.0383 | 0.77 |
| A0A2H6CJ58 | *groEL* | 60 kDa chaperonin | 0.0051 | 0.83 |
| A0A424XQ63 | *celF* | 6-phospho-beta-glucosidase | 0.0441 | 0.81 |
| A0A3G5FHD0 | *C7H83_03920* | Antitoxin | 0.0002 | 0.82 |
| A0A424XR69 | *C7K42_07545* | Arsenate reductase family protein | 0.0003 | 0.77 |
| G4L3Q0 | *pgmB* | Beta-phosphoglucomutase/glucose-1-phosphate phosphodismutase | 0.0382 | 0.82 |
| A0A3G5FK61 | *ftsX* | Cell division protein FtsX | 0.0382 | 0.81 |
| A0A424XSJ3 | *tyrA1* | Chorismate mutase | 0.0004 | 0.82 |
| A0A2H6CEN0 | *TEHD23766T_0879* | Cold shock protein | 0.0000 | 0.78 |
| A0A3G5FJM8 | *C7H83_08370* | Competence protein ComEA | 0.0015 | 0.83 |
| G4L6C9 | *copZ* | Copper chaperone CopZ | 0.0000 | 0.51 |
| A0A2H6DFT8 | *dtd* | D-aminoacyl-tRNA deacylase | 0.0473 | 0.72 |
| A0A6I5YI56 | *GLW17_05695* | Deoxyribonuclease I | 0.0007 | 0.79 |
| A0A3G5FHH4 | *dhaL* | Dihydroxyacetone kinase subunit L | 0.0033 | 0.68 |
| A0A2H6DY48 | *rbsD* | D-ribose pyranase | 0.0000 | 0.68 |
| A0A2H6DNY0 | *TEHN7121_0232* | DUF2179 domain-containing protein | 0.0001 | 0.59 |
| A0A3G5FFM5 | *C7H83_00750* | DUF2200 domain-containing protein | 0.0007 | 0.80 |
| A0A2H6D0P9 | *TEHN7121_1363* | GAF domain-containing protein | 0.0046 | 0.56 |
| A0A2H6CZX4 | *garD* | Galactarate dehydratase | 0.0105 | 0.76 |
| G4L4E8 | *gcsH* | GcvH family protein | 0.0035 | 0.82 |
| A0A2H6CSW3 | *nrdH* | Glutaredoxin-like protein NrdH | 0.0133 | 0.67 |
| A0A2H6CD22 | *gpo* | Glutathione peroxidase | 0.0000 | 0.64 |
| A0A424XRS2 | *C7K42_09565* | Glutathione-dependent disulfide-bond oxidoreductase | 0.0002 | 0.80 |
| A0A424XQW1 | *gcvH* | Glycine cleavage system protein H | 0.0023 | 0.82 |
| A0A6I5YDE1 | *GLW17_01900* | GNAT family N-acetyltransferase | 0.0253 | 0.79 |
| A0A6I5YHM4 | *GLW17_03350* | GNAT family N-acetyltransferase | 0.0001 | 0.79 |
| A0A6I5YMX3 | *GLW17_11030* | GNAT family N-acetyltransferase | 0.0048 | 0.80 |
| A0A3G5FH69 | *ltrA* | Group II intron reverse transcriptase/maturase | 0.0000 | 0.66 |
| A0A6I5YGV1 | *GLW17_02975* | IS110 family transposase | 0.0002 | 0.76 |
| A0A424XTJ0 | *C7K42_03230* | IS3 family transposase | 0.0016 | 0.70 |
| A0A2H6CQ13 | *ileS* | Isoleucine--tRNA ligase | 0.0124 | 0.79 |
| A0A2H6CK26 | *TEHN7121_0111* | Metallophos domain-containing protein | 0.0018 | 0.69 |
| A0A424XRU3 | *C7K42_09630* | N-acetyltransferase | 0.0000 | 0.80 |
| A0A2H6CCW9 | *opuCC* | Osmoprotectant ABC transporter permease protein | 0.0012 | 0.70 |
| A0A2H6DNF2 | *TEHN7118_0723* | Oxidoreductase | 0.0004 | 0.78 |
| A0A424XPU8 | *msrB* | Peptide methionine sulfoxide reductase MsrB | 0.0000 | 0.53 |
| A0A2H6D160 | *pgm* | Phosphoglucomutase | 0.0073 | 0.82 |
| G4L641 | *pgaM* | Phosphoglycerate mutase family protein | 0.0003 | 0.78 |
| A0A6I5YAQ4 | *ppaC* | Probable manganese-dependent inorganic pyrophosphatase | 0.0011 | 0.79 |
| A0A424XRT0 | *C7K42_08535* | Protein jag | 0.0000 | 0.74 |
| A0A2H6CE69 | *TEHD23766T_0709* | Pseudouridine synthase | 0.0007 | 0.74 |
| A0A2H6CDA3 | *deoD* | Purine nucleoside phosphorylase DeoD-type | 0.0398 | 0.83 |
| A0A2H6CC95 | *TEHD23766T_0037* | Putative ABC transporter ATP-binding protein | 0.0163 | 0.79 |
| A0A2H6CCF2 | *TEHD23766T_0095* | Putative acetyltransferase | 0.0200 | 0.67 |
| A0A2H6CE34 | *TEHD23766T_0675* | Putative acetyltransferase | 0.0017 | 0.81 |
| A0A2H6CT67 | *TEHN7118_0994* | Putative acetyltransferase | 0.0080 | 0.79 |
| A0A2H6CVA5 | *TEHN7118_1710* | Putative acetyltransferase | 0.0014 | 0.74 |
| G4L8X7 | *celC* | Putative beta-glucan-specific phosphotransferase system enzyme IIA component | 0.0286 | 0.82 |
| A0A2H6CGU1 | *TEHD23766T_1640* | Putative dipeptidase | 0.0047 | 0.78 |
| A0A2H6DAV0 | *TEHN7121_0357* | Putative iron-sulfur cluster assembly protein IscU | 0.0151 | 0.80 |
| A0A2H6CWJ8 | *TEHN7118_2168* | Putative L-cystine uptake protein | 0.0024 | 0.64 |
| G4L958 | *TEH_17550* | Putative membrane protein insertion efficiency factor | 0.0047 | 0.61 |
| A0A2H6C865 | *TEHN7118_0967* | Putative O-methyltransferase | 0.0023 | 0.83 |
| A0A2H6CW83 | *TEHN7118_2040* | Putative polysaccharide biosynthesis protein | 0.0123 | 0.82 |
| A0A2H6DP99 | *TEHN7118_1433* | Putative RpiR family transcriptional regulator | 0.0030 | 0.74 |
| G4L4R1 | *TEH_02840* | Putative Rrf2 family DNA-binding protein | 0.0005 | 0.75 |
| G4L478 | *TEH_24300* | Putative TetR family transcriptional regulator | 0.0002 | 0.79 |
| A0A2H6CIK1 | *TEHD23766T_2251* | Putative transposase | 0.0148 | 0.65 |
| G4L5M7 | *TEH_05240* | Putative Xre family DNA-binding protein | 0.0209 | 0.83 |
| A0A2H6CA37 | *TEHN7121_1400* | Putative Xre family DNA-binding protein | 0.0004 | 0.77 |
| A0A2H6CDG5 | *TEHD23766T_0455* | Putative Xre family DNA-binding protein | 0.0001 | 0.78 |
| A0A3G5FLI9 | *citC* | Pyridoxal phosphate homeostasis protein | 0.0001 | 0.73 |
| A0A6I5YCF8 | *GLW17_03120* | Restriction endonuclease subunit S | 0.0459 | 0.83 |
| A0A3G5FLC3 | *rsmH* | Ribosomal RNA small subunit methyltransferase H | 0.0046 | 0.75 |
| A0A2H6DSU1 | *rpoD* | RNA polymerase sigma factor SigA | 0.0000 | 0.78 |
| G4L6W6 | *ezrA* | Septation ring formation regulator EzrA | 0.0000 | 0.79 |
| A0A2H6CWU5 | *TEHN7121_0012* | TPM_phosphatase domain-containing protein | 0.0005 | 0.71 |
| A0A2H6CEM4 | *nusB* | Transcription antitermination protein NusB | 0.0031 | 0.79 |
| A0A2H6DC03 | *trhO* | tRNA uridine (34) hydroxylase | 0.0001 | 0.78 |
| A0A2H6CH25 | *xerD* | Tyrosine recombinase XerD | 0.0001 | 0.64 |
| A0A2H6C3Q8 | *TEHN7121_1101* | Uncharacterized protein | 0.0002 | 0.72 |
| A0A2H6CQI6 | *TEHN7118_0055* | Uncharacterized protein | 0.0385 | 0.83 |
| A0A2H6CUQ1 | *TEHN7118_1522* | Uncharacterized protein | 0.0113 | 0.82 |
| A0A3G5FIE3 | *C7H83_05885* | Uncharacterized protein | 0.0056 | 0.78 |
| G4L2L3 | *TEH_20150* | Uncharacterized protein | 0.0002 | 0.78 |
| G4L3P0 | *TEH_00290* | Uncharacterized protein | 0.0059 | 0.46 |
| G4L767 | *TEH_10640* | Uncharacterized protein | 0.0000 | 0.48 |
| A0A2H6CHI6 | *TEHD23766T_1884* | Uncharacterized protein | 0.0038 | 0.65 |
| A0A2H6CTN3 | *TEHN7118_1158* | Uncharacterized protein | 0.0000 | 0.67 |
| A0A2H6CW59 | *TEHN7118_2016* | Uncharacterized protein | 0.0142 | 0.80 |
| A0A2H6D0J7 | *TEHN7121_1299* | Uncharacterized protein | 0.0014 | 0.75 |
| A0A2H6D1P7 | *TEHN7121_1718* | Uncharacterized protein | 0.0124 | 0.82 |
| A0A2H6D966 | *TEHN7118_1747* | Uncharacterized protein | 0.0035 | 0.71 |
| A0A2H6DGX0 | *TEHN7118_1854* | Uncharacterized protein | 0.0002 | 0.76 |
| A0A2H6DVP6 | *TEHN7118_0496* | Uncharacterized protein | 0.0012 | 0.48 |
| G4L397 | *TEH_22460* | Uncharacterized protein | 0.0010 | 0.62 |
| G4L9H4 | *TEH_18710* | UPF0297 protein TEH_18710 | 0.0016 | 0.83 |
| A0A424XUT1 | *C7K42_05520* | XRE family transcriptional regulator | 0.0066 | 0.82 |
| A0A424XT28 | *C7K42_02225* | YbaK/EbsC family protein | 0.0127 | 0.81 |
| A0A424XT36 | *C7K42_02290* | YibE/F family protein | 0.0124 | 0.68 |
| A0A3G5FGW7 | *rpmF* | 50S ribosomal protein L32 | 0.0383 | 0.77 |
| A0A2H6CJ58 | *groEL* | 60 kDa chaperonin | 0.0051 | 0.83 |
| A0A424XQ63 | *celF* | 6-phospho-beta-glucosidase | 0.0441 | 0.81 |
| A0A2H6D3J1 | *mutY* | Adenine DNA glycosylase | 0.0075 | 0.83 |


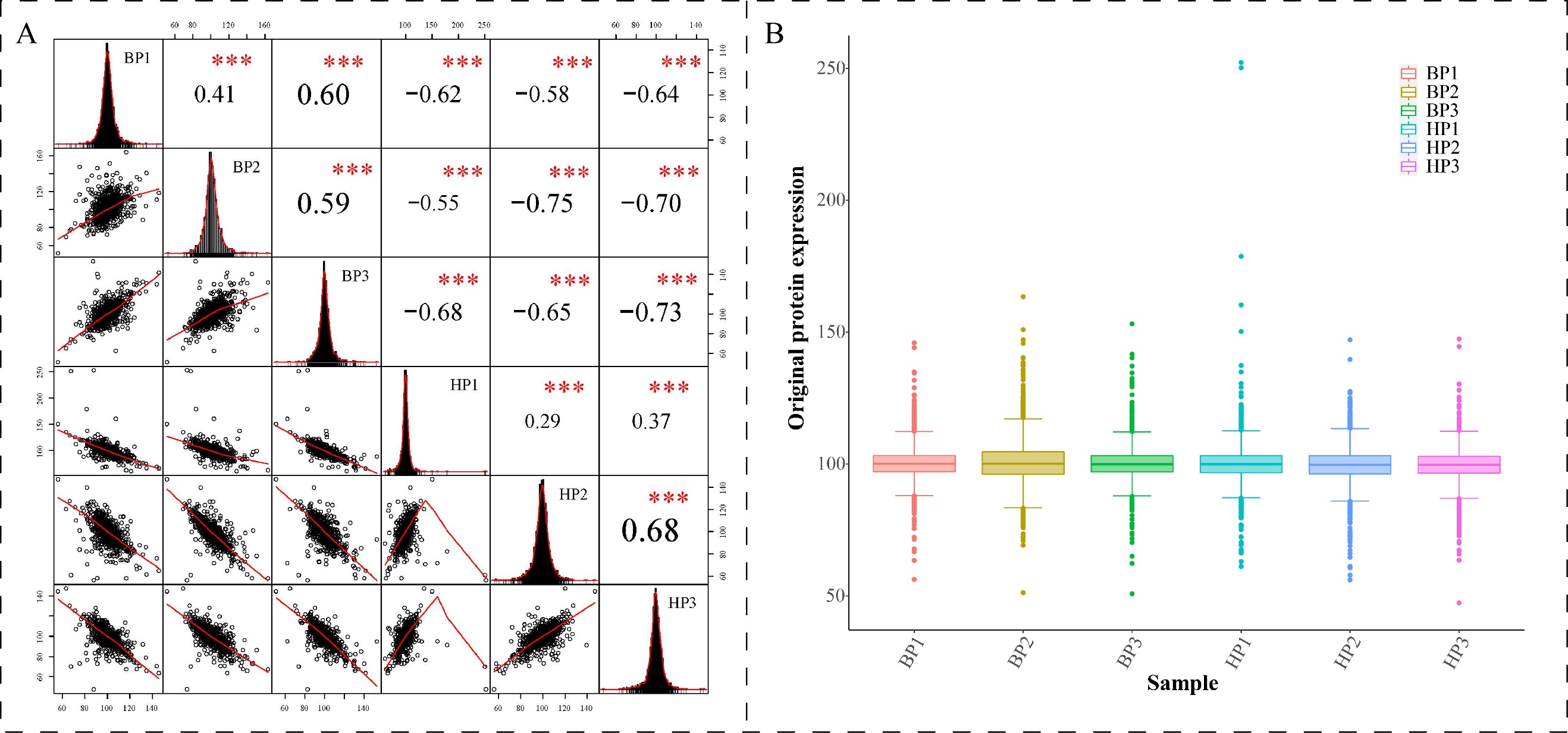


**Figure S1**





**Figure S2**
